# Supplementary material for: Transcriptome-driven constraint-based modelling reveals metabolic targets for ovarian cancer
Source: Cancer Metab. 2026 Mar 19;14:7. doi: 10.1186/s40170-026-00425-6 (PMC13003690; doi:10.1186/s40170-026-00425-6)
Supplement: Supplementary file 2 — Supplementary Material 2 [file 40170_2026_425_MOESM2_ESM.docx]

**Supplementary data**

| **Cell line model** | **p-value** | **r** |
| --- | --- | --- |
| **COV504** | 7.6398 x 10^-22^ | -0.498762 |
| **SNU8** | 6.6205 x 10^-52^ | -0.468450 |
| **JHOS2** | 3.00298 x 10^-31^ | -0.452691 |
| **PEA1** | 1.63158 x 10^-97^ | -0.404164 |
| **59M** | 6.41651 x 10^-10^ | -0.368615 |
| **OAW28** | 9.09970 x 10^-79^ | -0.355036 |
| **PEO4** | 5.18932 x 10^-9^ | -0.183914 |
| **CAOV3** | 2.68982 x 10^-5^ | -0.173970 |
| **JHOS4** | 4.09742 x 10^-4^ | -0.165705 |
| **ES2** | 8.57693 x 10^-10^ | -0.139105 |
| **PEO1** | 2.68663 x 10^-9^ | -0.130448 |
| **CAOV4** | 2.94089 x 10^-10^ | -0.119685 |
| **NIHOVCAR3** | 0.0452459 | -0.117682 |
| **COV318** | 0.1400247 | -0.100973 |
| **HEYA8** | 0.6149733 | -0.028539 |
| **KURAMOCHI** | 0.6070811 | 0.012544 |
| **COV362** | 0.3106583 | 0.023102 |

**Supplementary Table 1. Pearson correlation and p-values for gene knockdown simulations.** The 13 out of 17 significant models are those below with p≤0.05. Ordered from smallest to largest r.

| **Dataset** | **General description** | **Normalisation and processing details** | **Reference and accession** |
| --- | --- | --- | --- |
| **CCLE transcriptomics** | 64 ovarian cell lines;  53,970 genes | RSEM; Log_2_(TPM+1) | CCLE_expression.csv; Depmap |
| **CCLE CRISPR-Cas9 gene dependency dataset** | 1,086 total cell lines; 17,387 genes |  | CRISPR_gene_dependency.csv; Depmap |

**Supplementary Table 2. Description of input and validation datasets.** RSEM: RNAseq by expectation-maximisation; TPM: transcripts per million. Cell line annotations including source of experimental proliferation rate and optimal media conditions, which were replicated in silico, have been described in the Supplementary file ‘CCLE_2020_transcriptomics_annotations.xlsx’.

| **Cell line** | **Experimental cell culture conditions** | **Seeding dilution into new flask** |
| --- | --- | --- |
| **A549** | RPMI1640 + 10% fetal bovine serum (FBS) + 50 µg/mL penicillin/streptomycin | 1:6 |
| **OV56** | 1:1 ratio of Dulbecco’s Modified Eagle’s Medium (DMEM) and Ham’s F-12 nutrient mix + 5% FBS + 2 mM glutamine + 0.5 μg/mL hydrocortisone (Sigma Aldrich) + 10 μg/mL insulin (Sigma Aldrich) + 50 μg/mL penicillin/streptomycin | 1:8 |

**Supplementary Table 3. Experimental conditions for cell culture.** All media and supplements are from Life Technologies.

| Target gene | Manufacturer details | Sequences |
| --- | --- | --- |
| TPI1 | ON-TARGETplus Human TPI1 siRNA  Cat # L-009776-00-0005 | 1. GAGCCUGUGUGGGCCAUUG 2. CCAGGAAGUACACGAGAAG 3. GGGUGGUGCUUCCCUCAAG 4. GCAGAAAGUGGCCCAUGCU |
| MYC | ON-TARGETplus MYC siRNA oligonucleotide #4  Cat# J-003282-26  (Topham et al., 2015) | CGAUGUUGUUUCUGUGGAA |
| Non-targeting control pool | ON-TARGETplus Non-targeting Control Pool  Cat# D-001810-10 | 1. UGGUUUACAUGUCGACUAA 2. UGGUUUACAUGUUGUGUGA 3. UGGUUUACAUGUUUUCUGA 4. UGGUUUACAUGUUUUCCUA |

**Supplementary Table 4. Details of siRNA sequences.**


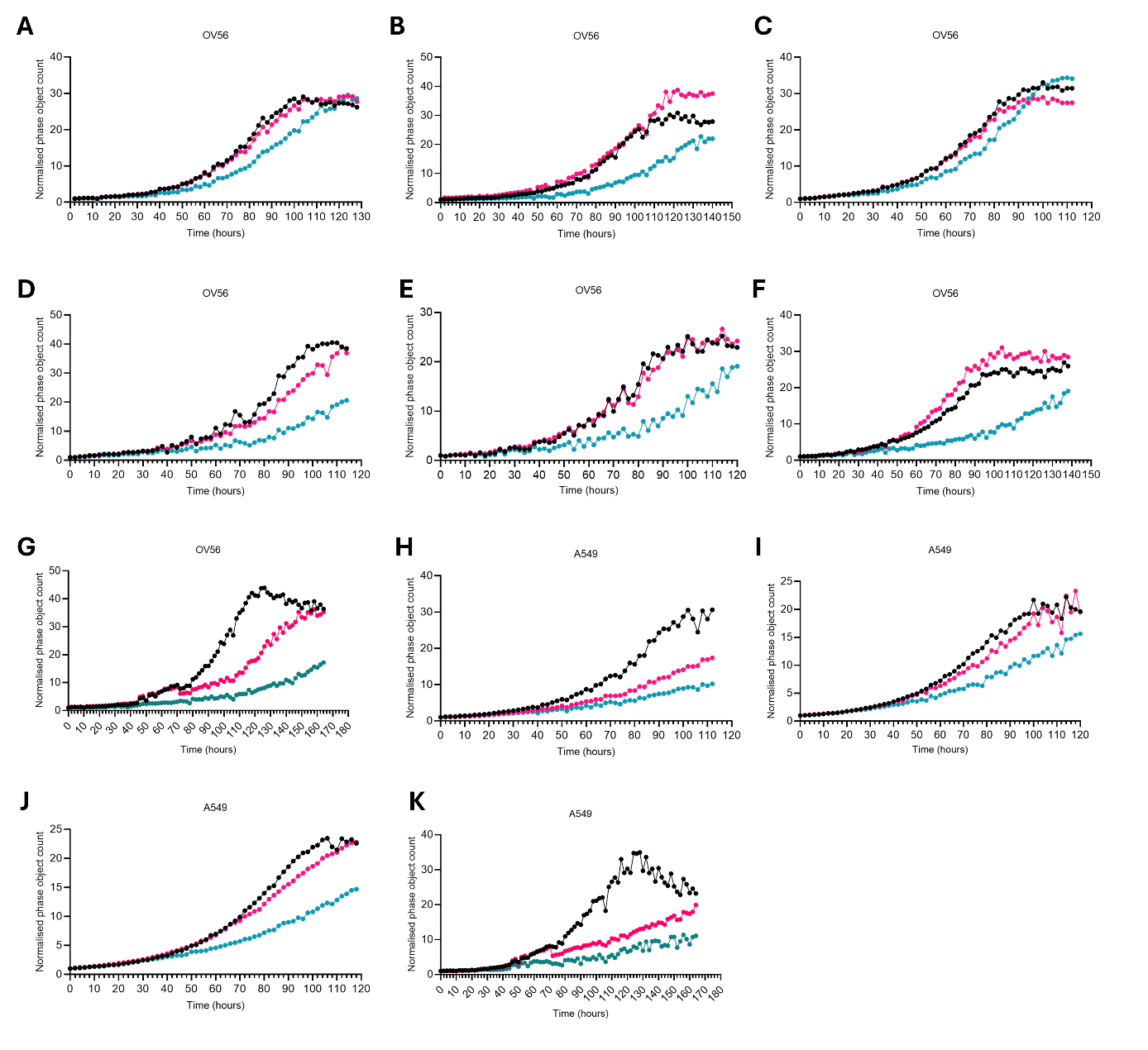


**Supplementary Figure 1. Biological repeats for the siRNA transfection of OV56 and A549.**

**A-G** Proliferation of OV56 cells upon siRNA transfection (NTsiRNA: black, siTPI1: pink, siMYC: teal). Live cell microscopy assay, using the Incucyte S3^®^ live-cell analysis system (Sartorius). **H-K** Proliferation of A549 cells upon siRNA transfection (NTsiRNA: black, siTPI1: pink, siMYC: teal). Live cell microscopy assay, using the Incucyte S3^®^ live-cell analysis system (Sartorius). Associated with Figure 3, showing biological replicates for the siRNA transfection of OV56 and A549 cell lines. The growth effect, as calculated using the normalised phase object count at 100 hours for siTPI1 transfection compared to NTsiRNA transfection has been included in Figure 3F.
